# Prolonged response with bevacizumab in a patient with benign metastasizing leiomyomatosis

**DOI:** 10.1016/j.gore.2021.100903

**Published:** 2021-12-18

**Authors:** Shalini Chhabra, Pooja Sangani, Reagan Saig, Michael Stany

**Affiliations:** a Graves Gilbert Clinic, Bowling Green KY, United States; b Department of Obstetrics and Gynecology, St. Thomas Midtown Hospital, Nashville, TN, United States; c University of Tennessee Health Science Services, Department of Gynecologic Oncology, St. Thomas Midtown Hospital, Nashville, TN, United States

## Abstract

• Benign metastasizing leiomyomatosis (BML) is a rare condition.
• Treatment options have traditionally included surgical resection or hormonal based therapy.
• Traditional chemotherapy for BML is ineffective.
• Our patient has had a prolonged response of stable disease with the anti-VEGF therapy of bevacizumab.

## 1 Introduction

Benign metastasizing leiomyomatosis (BML) is a rare condition seen mostly in pre-menopausal women who have been treated for uterine leiomyoma with myomectomy or hysterectomy. Smooth muscle tumors metastasize most commonly to the lungs, abdomen, and pelvis, or they can be intravascular. Over 200 cases have been reported since Dr. Steiner initially described BML in 1939 (Steiner, 1939). Patients usually present after their third decade of life (Vaquero et al., 2009). There is an average interval of 10 years from the initial uterine surgery to diagnosis of BML. BML is mostly asymptomatic, hence discovered on routine examination or when metastasized. Due to the nature of the tumor being mostly asymptomatic, it can metastasize to the lungs before it is detected.

As a rare disease, there is a lack of understanding of its pathology and method of spread. BML has been treated with surgical resection, as well as anti-estrogen therapies like aromatase inhibitors and GnRH agonists ([Shozu M, Sumitani H, Segawa T, Yang H-J, Shimada K. In Situ expression of Aromatase P450 in Leiomyoma, of the uterus by Leuprolein, acetate. J of Clin endocrinol Metab, 2001). Because tumors are not very mitotically active, chemotherapy is considered ineffective. We report a patient who now has a prolonged response of stable disease with the vascular endothelial growth factor (VEGF) monoclonal antibody bevacizumab.

## 2 Case report

**Fig. 1:**
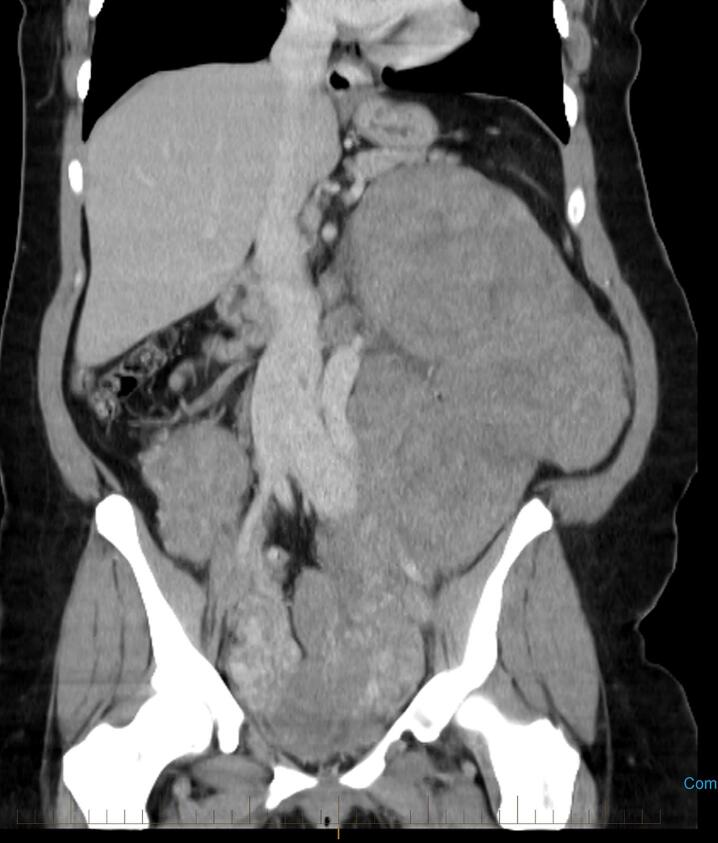
CT scan obtained at presentation, coronal view demonstrating a mass spanning from the pelvis to left upper quadrant.

A 38-year-old female presented with pelvic pain three years after undergoing a robotic hysterectomy for fibroids. A CT scan showed a large 20 cm abdominopelvic mass, pulmonary nodules, and an intra-caval tumor thrombus (Fig. 1). A pelvic MRI showed multiple confluent masses throughout the abdominopelvic cavity extending from the cul-de-sac to the mid-abdomen. CT guided biopsies of the pelvic and lung masses showed spindle cell tumor histology that were ER positive. Immunohistochemical staining for desmin A was strongly positive, consistent with a smooth muscle origin. Ki 67 demonstrated a mitotic rate of less than 5 %. No cytologic atypia or tumor cell necrosis was noted. These radiologic and histologic findings confirmed the diagnosis of benign metastasizing leiomyomatosis.

Dual anti-estrogen therapy with IM leuprolide and letrozole 2.5 mg orally daily was started. As the pelvic mass was too large for resection with multiple collateral feeder vessels, the patient underwent mass embolization by interventional radiology. After three months of combination hormonal treatment and four total embolizations, her CT scan showed increasing size of her pulmonary lesions while the abdominal mass and IVC thrombus did not change.

Given the progression of her pulmonary lesions, she was referred to the National Institutes of Health where she was treated with ulipristal acetate15 mg orally daily, a selective progesterone receptor modulator. Unfortunately, disease progression occurred after 4 months of treatment.

As the patient had received hormonal treatments that target both the estrogen and progesterone receptors, the decision was made to start bevacizumab, targeting angiogenesis. To avoid any estrogen stimulation of the tumors, both leuprolide and letrozole were continued. After three cycles of treatment with bevacizumab 15 mg/kg every 3 weeks, the CT scan showed a slight decrease in one of the pelvic masses with stable disease of all other pulmonary and abdominal masses. Hypertension was the main toxicity that was managed successfully with medication. After approximately 30 cycles, treatment intervals were then increased to every 4 weeks given the long half-life of bevacizumab and trying to minimize toxicities with long term use. Currently, the patient has received over 40 cycles of bevacizumab with stable disease of all lesions with acceptable toxicity.

## 3 Discussion

As a rare disease, the treatment of BML is challenging and not standardized. If lesions are resectable, surgery is generally felt to be the most reasonable initial treatment. When systemic treatment is needed in cases where the leiomyomas cannot be resected, estrogen deprivation therapies are generally first line. Multiple hormonal therapies have been described (Table 1). BML tumors have been treated with antiestrogen therapy since they were found to be estrogen receptor (ER) positive by Cramer et al in 1980 (Cramer et al., 1980).

**Table 1:** Treatment options for BML-Case reports showing different treatments used for treating BML.

Author & Year of Publication	Number of patients	Treatments	Results/Duration
Raposo et al. 2018 (6)	2	Aromatase Inhibitor	Complete – 9 months Stable disease – 6 months
Pastre et al. 2017 (13)	1	Aromatase inhibitor	Complete – 45 months
Efared et al. (2017) (14)	1	Aromatase inhibitor	Stable disease – 6 months
Findakly et al 2020 (15)	1	Leuprolide and aromatase inhibitor	Stable disease – 5 years

Abbreviations: BML-Benign metastasizing leiomyomatosis.

GnRH agonist treatment of BML, first described by Hague et al (Hague et al., 1986) in 1986, suppresses the pituitary-ovarian axis of estrogen production. Aromatase inhibitors (anastrozole and letrozole) have also been used for treatment of BML (Raposo et al., 2018). Leiomyomas have been found to over-express aromatase P450 (Sanci et al., 2011). Therefore, aromatase inhibition may work at the peripheral level by decreasing estrogen levels and locally in leiomyomas.

The literature has reported variable responses with progesterone therapy, with some reports citing worsening of symptoms (Cramer et al., 1980) while others showing regression of disease and symptomatic relief (Jautzke et al., 1996). The different responses to progesterone treatment may be explained by the fact that progesterone can increase both Bcl-2, an apoptosis-inhibiting gene product, and tumor necrosis factor-alpha, a cytokine that induces apoptosis (Maruo et al., 2003).

Cytotoxic treatments are ineffective against BML. Reports of BML tumors resected after chemotherapy have shown no signs of necrosis, thrombosis, or fibrosis on pathologic exam highlighting the lack of any treatment effect (Ottlakan et al., 2016). Histologically, BML tumors lack the diagnostic criteria for leiomyosarcoma that includes a high number of mitoses. Cytotoxic treatment targets cells during cell cycle division, and since leiomyoma cells are not rapidly dividing, they are not sensitive to this treatment.

While leiomyomas may not be rapidly growing tumors, multiple studies have shown that angiogenic factors are upregulated when compared to myometrium (Tal and Segars, 20142014). BML tumors lack the typical aggressive characteristics of cancer but have a propensity for continued growth. BML is likely on the smooth muscle tumor spectrum that ranges from intrauterine fibroids to leiomyosarcoma.

Angiogenic factors that include VEGF have been found to be important in the pathogenesis of leiomyosarcomas (Sumitani et al., 2000). Given the importance of angiogenesis, therapy that targeted this pathway was initiated after 7 months when traditional hormonal treatment failed. After 3 years of treatment with bevacizumab, the patient continues to have stable disease. This highlights the importance of angiogenesis in BML. Bevacizumab and other therapies targeting angiogenesis may be treatment options in patients with BML.

## Consent

Informed consent was obtained from the patient presented in this case report.

### CRediT authorship contribution statement

**Shalini Chhabra:** Writing – original draft. **Pooja Sangani:** Data curation, Investigation. **Reagan Saig:** Data curation, Writing – review & editing. **Michael Stany:** Conceptualization, Supervision, Writing – review & editing.

## Declaration of Competing Interest

The authors declare that they have no known competing financial interests or personal relationships that could have appeared to influence the work reported in this paper.
